# Laparoscopic ileo-transverse bypass may contribute to achieving curative resection for locally advanced right colon cancer: a case report

**DOI:** 10.1186/s40792-021-01221-8

**Published:** 2021-06-02

**Authors:** Shunsuke Tabe, Toru Tonooka, Isamu Hoshino, Nobuhiro Takiguchi, Hiroaki Soda, Hisashi Gunji, Yoshihiro Nabeya, Masayuki Ohtsuka

**Affiliations:** 1 Department of Gastroenterological Surgery, Chiba Cancer Center, 666-2 Nitonacho, Chuo-ku, Chiba, 260-8717 Japan; 2 Department of General Surgery, Chiba University Graduate School of Medicine, 1-8-1 Inohana, Chuo-ku, Chiba, 260-8677 Japan

**Keywords:** Laparoscopic ileo-transverse colon bypass, Obstructive colon cancer, Preoperative chemotherapy

## Abstract

**Background:**

The strategy for treating obstructive colon cancers with metastatic lesions remains unclear. Herein, we report a case of laparoscopic ileo-transverse colon bypass (LITB) before preoperative chemotherapy for an obstructive right colon cancer.

**Case presentation:**

A 59-year-old woman was referred to our institution (Department of Gastroenterological Surgery, Chiba Cancer Center) for liver tumors detected on ultrasound. The clinical diagnosis was ascending colon cancer with multiple liver metastases. Based on the criteria of the International Union against Cancer Committee, 8th edition, the staging was confirmed as cT4aN1M1a(H), cStage IV. Although the primary tumor in the ascending colon extended beyond the colonic wall, curative resection was possible for both primary and metastatic tumors. We planned to administer chemotherapy before the radical surgery to obtain tumor-free resection margins; however, as the obstruction was fatal, LITB was prioritized and performed using five ports. An intracorporeal side-to-side anastomosis was performed between the ileum, 25 cm from the terminal ileum, and the transverse colon. The patient was discharged on postoperative day 18 without any complications. After LITB, for preoperative chemotherapy, five courses of capecitabine plus oxaliplatin (CapeOX) + bevacizumab were administered. Six weeks after the preoperative chemotherapy, right hemicolectomy with D3 lymph node dissection and right hepatectomy were performed. Pathological findings of the resected specimen confirmed curative resection of both lesions, and a favorable effect of chemotherapy was obtained. The patient has been alive for over 8 months after the surgery, with no evidence of cancer recurrence.

**Conclusions:**

This case report demonstrates the effectiveness of LITB for obstructive right colon cancer in patients who need preoperative chemotherapy.

## Background

Currently, there are many treatment strategies for obstructive advanced colon cancers. Resection of the primary tumor, ileostomy or colostomy, and ileo-colon bypass are various surgical approaches. Furthermore, trans-anal ileus tube and colonic stent placement may be selected as alternative therapies. According to the Japan Colorectal Cancer Society (JSCCR) Guideline 2019 [1], resection should be the first choice for resectable advanced colon cancers even with metastases to other organs. The efficacy and safety of preoperative chemotherapy for cases with resectable liver metastasis have not been established; however, in cases of locally advanced colon cancers involving other organs, surgery without preoperative chemotherapy decreases the curative resection rate and increases perioperative complications [2]. In Europe, clinical trials (FOxTROT [3] and PRODIGE 22 [4]) investigating the efficacy of preoperative chemotherapy for locally advanced colorectal cancers without distant metastasis have been conducted recently and the oncological outcomes are expected soon. Although the evidence supporting preoperative chemotherapy is insufficient, this strategy may contribute to increased R0 resection rates for locally advanced colon cancers that involve other organs.

Herein, we report a case of laparoscopic ileo-transverse bypass (LITB) surgery for obstructive right colon cancer (ORCC) with liver metastasis. After the bypass was established, preoperative chemotherapy was administered, and radical resection of the primary lesion and metastatic area was performed. An ileo-transverse bypass is often performed as a palliative therapy for a non-curative ORCC. Several case reports of such a bypass surgery have been published in Japan; however, these patients have not been treated aggressively because they did not have a good performance status. Therefore, we have included LITB as a curative treatment strategy and administered molecular-target drugs to obtain tumor shrinkage of the primary and metastatic lesions. By combining these therapies, we could achieve curative resection for advanced colon cancer. To our knowledge, there have been no reports of including bypass therapy as an aggressive treatment in such cases; thus, our case report is the first of its kind.

## Case presentation

**Fig. 1:**
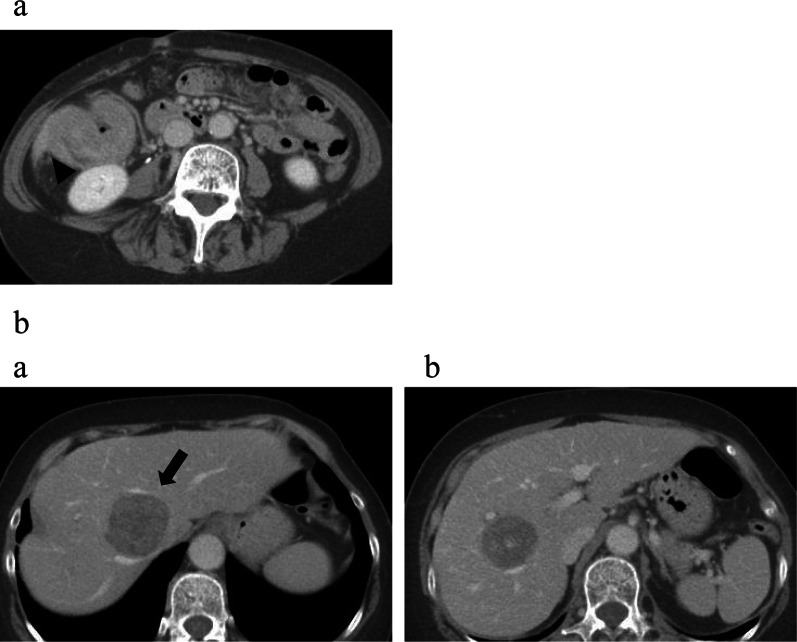
Computed tomography images before preoperative chemotherapy. **a** An enhanced mass in the ascending colon is seen invading the abdominal wall and the inferior edge of the liver (black arrowhead). **b** (**a**) The ventral lesion is seen touching the middle hepatic vein (black arrow). (**b**) The dorsal lesion of hepatic metastasis

**Fig. 2:**
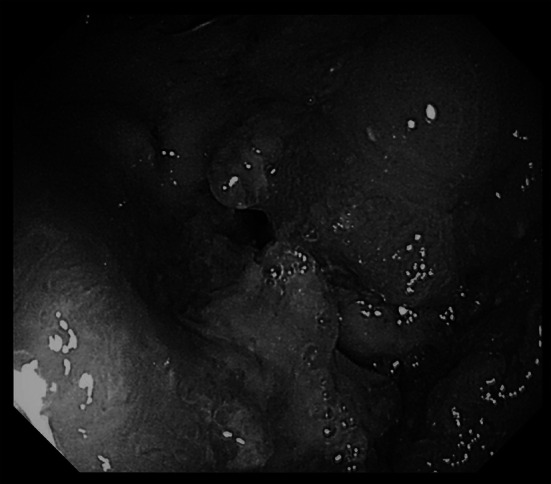
Colonoscopy shows a circumferential tumor in the ascending colon, and the fiber was unable to pass beyond it

A 59-year-old woman was referred to our institution for liver tumors detected on ultrasound. Computed tomography (CT) revealed an enhanced mass in the ascending colon that invaded the abdominal wall and the inferior edge of the liver. The regional lymph nodes (#202, #212, and #221) were distinctly swollen and suspected to be metastatic. There were two metastatic lesions in liver segment 8, and the hilar lymph nodes were also swollen (Fig. 1a, b). Colonoscopy showed a circumferential tumor in the ascending colon, and the scope was unable to pass beyond it (Fig. 2). The tumor marker levels were as follows: carcinoembryonic antigen, 13.7 (upper reference limit, 5.0 ng/dL); carbohydrate antigen 19-9, 2.64 U/mL (upper reference limit, 37.0 IU/mL). Other laboratory data revealed abnormal values of γ-guanosine triphosphate (41 U/L; reference range, 9–32 U/L) and alkaline phosphatase (397 U/L; reference range, 106–322 U/L); levels of all other investigations were within the normal range. Biopsy findings from the primary tumor revealed an adenocarcinoma (tub1 > tub2) and the RAS pattern was mutant. Based on the criteria of the International Union against Cancer Committee (UICC, 8th edition), the diagnosis was ascending colon cancer with multiple liver metastases and the clinical stage was cT4aN1M1a(H), cStage IV.

**Fig. 3:**
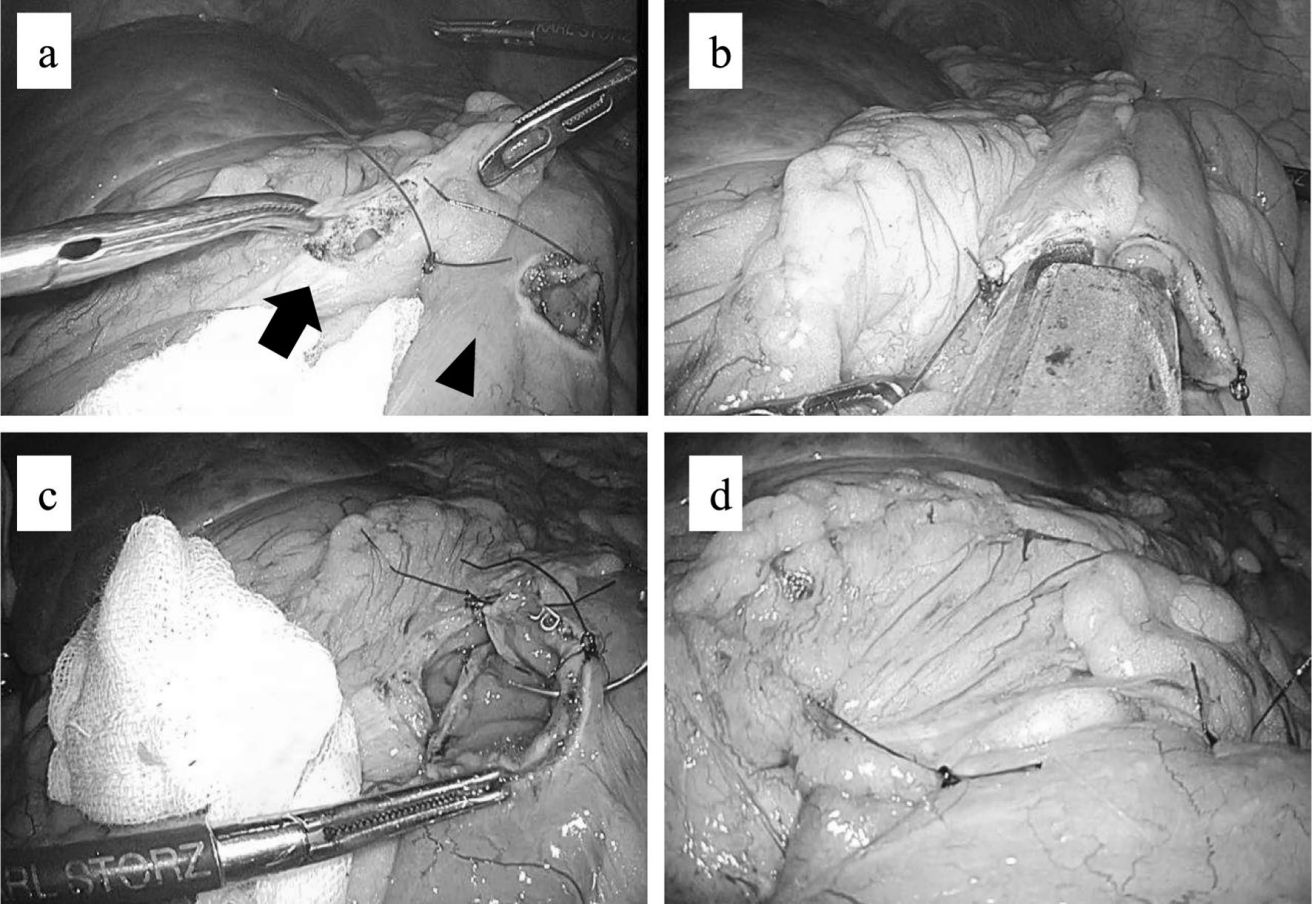
Images of the intracorporeal anastomosis technique. **a** The terminal ileum and transverse colon were placed side by side, and each entry hole was made (black arrow: transverse colon; black arrowhead: ileum). **b** A side-to-side anastomosis was created by inserting linear staplers into the entry holes. **c** The entry hole of the staplers was closed by hand-sewn fashion by the Albert–Lembert method. **d** Laparoscopic ileo-transverse colon bypass was accomplished

The patient required curative resection, but as the primary tumor in the colon extended beyond the colonic wall, we decided to administer preoperative chemotherapy before the radical surgery to obtain free radial resection margins. However, as the obstruction was severe, LITB was performed as the initial treatment. LITB was initiated with five ports after confirming the absence of peritoneal dissemination. Subsequently, part of the ileum, 25 cm from the terminal ileum, and the transverse colon were placed together for side-to-side anastomosis. The anastomosis was constructed using linear staplers (Signia Endo-GIA TM, purple 60 mm) after placement of three stay sutures. To prevent peritoneal dissemination and intra-abdominal infection, we covered the stapler device after anastomosis, and the entry hole of the staplers was closed in a hand-sewn fashion using the Albert–Lembert method (Fig. 3).

The surgical time was 98 min, and the blood loss was 5 mL. The patient was discharged on postoperative day (POD) 18 without complications.

**Fig. 4:**
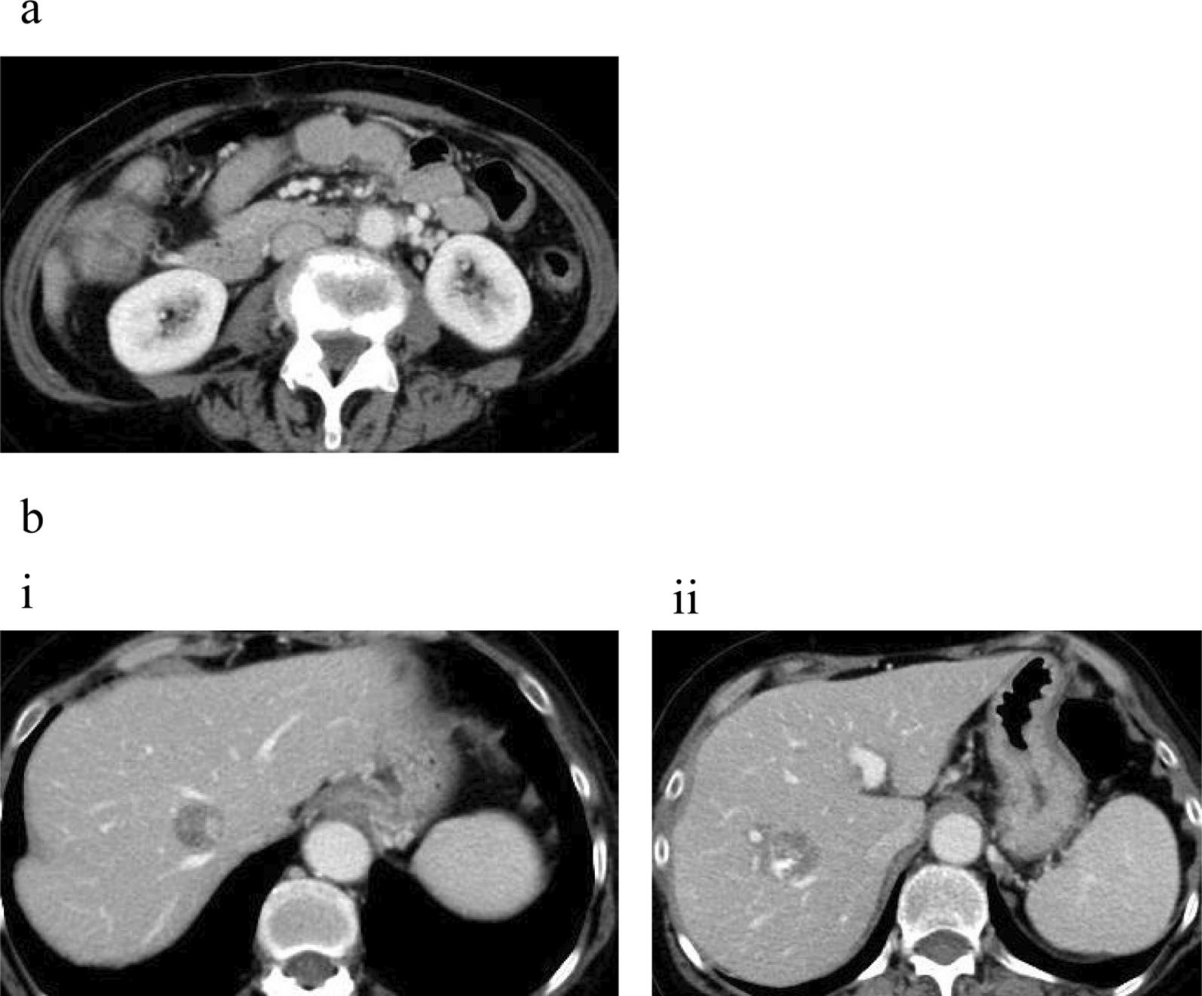
Computed tomography images after preoperative chemotherapy**.** **a** Shrinkage of the primary tumor is seen. **b** (**i**), (**ii**) Shrinkage of the two metastatic lesions in the liver is seen

Four weeks after the first surgery, preoperative chemotherapy, capecitabine plus oxaliplatin (CapeOX) + bevacizumab, was administered. Five courses of chemotherapy were administered, although a reduction in capecitabine dose was required due to stomatitis (Common Terminology Criteria for Adverse Events Grade 1). CT after preoperative chemotherapy revealed a shrinkage of the primary tumor and metastatic lesion (Fig. 4a, b), although the tumor marker levels remained unchanged. Six weeks after the preoperative chemotherapy, right hemicolectomy with D3 lymph node dissection and right hepatectomy were performed, based on the diagnosis of ascending colon cancer with multiple liver metastases (ycT4aN1M1, ycStage IV). First, the surgery was performed from the liver part, and when the dissection of the liver was complete, the operation was performed from the colon part. Since a side-to-side anastomosis had been performed in the previous bypass surgery, right hemicolectomy was simply completed by resecting the ileum and transverse colon after complete mesocolic excision. The surgical time for colectomy was 90 min, and the blood loss was 30 mL; the total surgical time was 382 min, and the total blood loss was 530 mL. Temporary chylous ascites appeared after the operation, but improved with observation. The patient was discharged from the hospital on POD 15 without complications.

**Fig. 5:**
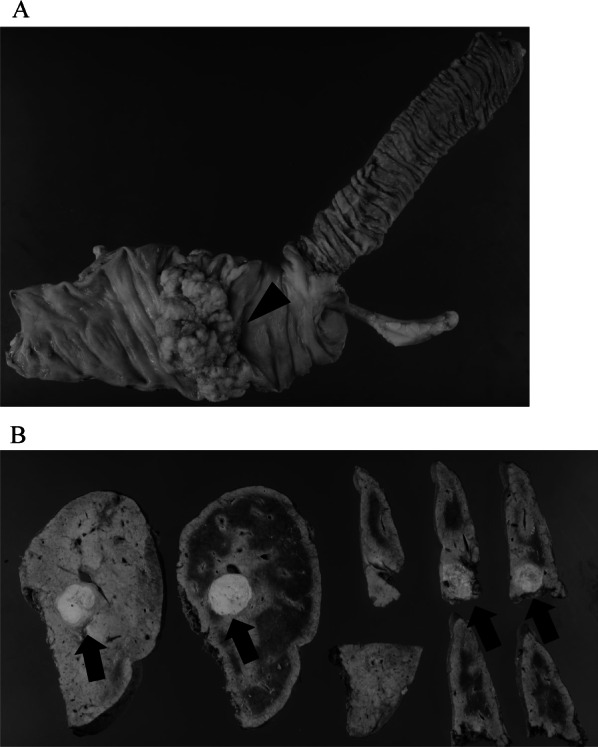
Pathological findings**.** **A** Resected specimen of the ascending colon tumor shows a moderately differentiated adenocarcinoma with negative resection margins (black arrowhead). **B** The two lesions at segment 8 in the liver show metastatic adenocarcinoma with negative resection margins (black arrow)

Pathological findings of the resected specimen confirmed that the ascending colon tumor was a moderately differentiated adenocarcinoma with negative resection margins. The effectiveness of chemotherapy was Grade 1a (Japanese Society for Cancer of the Colon and Rectum, 9th edition). Four lesions in segment 8 of the liver were confirmed as metastatic adenocarcinoma with negative resection margins, and the effectiveness of chemotherapy was Grade 2 (Fig. 5A, B). The final pathological diagnosis was ypT3N0M1a(H1), ypStage IVa. After radical operation, the patient did not receive chemotherapy due to liver dysfunction and ascites. She has been alive for 8 months after the surgery, with no evidence of cancer recurrence.

## Discussion

For treating stage IV colon cancer, resection is preferred if both primary and metastatic tumors are resectable [1]. The clinical guidelines recommend resection of a primary tumor with severe symptoms (bleeding, anemia, perforation, obstruction, etc.), regardless of whether the metastatic lesion is resectable. There are few reports of bypass surgery for advanced right colon cancers because advanced colon cancers can be resected only when there is no invasion of other organs (duodenum or liver). LITB is performed as a palliative therapy for ORCCs. Ida et al. [5] reported three cases of ileo-transverse bypass as a palliative therapy. There is no previous report of patients undergoing LITB prior to preoperative chemotherapy. Since laparoscopic bypass surgery is less invasive and causes less postoperative adhesions, it is effective when performed before a radical surgery for ORCC.

Currently, the Japanese guidelines for colon cancer treatment do not recommend preoperative chemotherapy for resectable colon cancers, and resection is performed as the first treatment if the metastatic lesions can be resected even in advanced colon cancers with liver metastases. This treatment policy is based on the EORTC 40983 trial [6]. Moreover, a majority of the experts in Japan suspect the efficacy of preoperative chemotherapy for colon cancers. Meanwhile, a few phase II trials investigating the efficacy of preoperative chemotherapy for advanced colorectal cancers have been reported in Europe and are drawing attention. The first is the FOxTROT trial [3] comprising 150 patients with resectable locally advanced colon cancers (TNM stage T3–4, N0–2, M0). This trial compared groups of patients who received three courses of preoperative chemotherapy (5-fluorouracil, leucovorin, and oxaliplatin [FOLFOX]) prior to radical surgery with those who received surgery first. The results showed that the lymph node metastasis rate and the R1 or R2 resection rate in the preoperative chemotherapy group was lower than that in the surgery first group. There were no significant differences in perioperative complications and mortality between the two groups. This trial concluded that preoperative chemotherapy for locally advanced colon cancers may contribute to a reduction in the locoregional recurrence rate and may downstage the primary tumor. The second is the PRODIGE 22 trial [4] comprising 120 patients with resectable colon cancers (TNM stage T3–4, and/or N2, M0). This trial also reported a reduction and downstaging of the primary tumor in the group that received four courses of preoperative chemotherapy (FOLFOX). As both these studies are phase II trials and the patient groups has no distant metastasis, there is no strong evidence to support their results; however, future studies should reconsider the efficacy of preoperative chemotherapy in resectable advanced colon cancers. In addition, it must be noted that some patients deviated from the protocol due to tumor progression and some postponed the surgery due to adverse events in the preoperative chemotherapy group. Thus, it is important to identify appropriate patients for preoperative chemotherapy.

In the present case, the metastatic lesions were resectable, but the primary lesion was borderline resectable due to tumor extension to the abdominal wall and liver. We judged that preoperative chemotherapy was necessary to achieve curative resection. Pathological findings revealed that the resected margins of the primary tumor and metastatic lesions were negative, with no tumor exposure in any area. The effectiveness of chemotherapy was Grade 1a at the primary site and Grade 2 at the liver metastatic sites, and preoperative chemotherapy may have contributed to achieving curative resection.

Since LITB is a laparoscopic and intracorporeal method, it is less invasive and beneficial for ORCC. Self-expandable metallic colon stents (SEMS) are less invasive and safe for older patients with poor general condition and multiple organ resection [7]. However, the use of monoclonal antibody drugs (e.g., bevacizumab) in patients receiving SEMS limits the choice of chemotherapy because of the possibility of perforation of the intestinal tract [8]. Ileostomy for ORCC could be selected as palliative therapy, but the management of the ileal stoma sometimes leads to complications such as high output syndrome [9]. In our case, LITB was performed before initiating preoperative chemotherapy with bevacizumab. Recently, laparoscopic palliative surgery for colorectal cancers has been frequently performed [10, 11] to reduce postoperative pain and shorten the hospital stay. However, the risk of peritoneal dissemination and intra-abdominal infections after intracorporeal anastomosis require attention after a laparoscopic bypass surgery. Sun et al. reported that, compared with extracorporeal anastomosis, intracorporeal anastomosis increases the possibility of intra-abdominal infections in patients who undergo right hemicolectomy [12]; therefore, prevention of this complication should be taken into consideration.

## Conclusions

We report the first case including bypass therapy as an aggressive treatment for ORCC. Although the efficacy and safety of this preoperative therapy have not been established, our case demonstrates that LITB and preoperative chemotherapy may contribute to achieving curative resection for locally advanced borderline resectable colon cancers.

## Data Availability

Not applicable.

## Abbreviations

LITB: Laparoscopic ileo-transverse bypass
ORCC: Obstructive right colon cancer
UICC: Union for International Cancer Control
POD: Postoperative day
CapeOX: Capecitabine plus oxaliplatin
FOLFOX: 5-Fluorouracil, leucovorin, and oxaliplatin
